# NFAT3 and TGF-β/SMAD3 regulate the expression of miR-140 in osteoarthritis

**DOI:** 10.1186/ar4387

**Published:** 2013-11-21

**Authors:** Ginette Tardif, Jean-Pierre Pelletier, Hassan Fahmi, David Hum, Yue Zhang, Mohit Kapoor, Johanne Martel-Pelletier

**Affiliations:** 1 Osteoarthritis Research Unit, University of Montreal Hospital Research Centre (CRCHUM), Notre-Dame Hospital, 1560 Sherbrooke Street East, Montreal, Quebec H2L 4M1, Canada

## Abstract

**Introduction:**

MicroRNAs (miRNAs) down-regulate their target genes. The intronic miR-140, present in the WW domain containing E3 ubiquitin protein ligase 2 (*WWP2*) gene, decreases the expression of genes that play detrimental roles in osteoarthritis (OA). As the expression level of miR-140 is significantly decreased in human OA chondrocytes, we investigated its regulation in those cells.

**Methods:**

Gene expression in human chondrocytes was determined by quantitative polymerase chain reaction (qPCR) and gene silencing was done in OA chondrocytes by transient transfection with specific small interfering RNAs (siRNAs). Binding sites of the miR-140 regulatory sequence (rsmiR-140) were identified by mutagenesis and chromatin immunoprecipitation (ChIP) in OA chondrocytes. The effects of translocation on OA chondrocytes were determined by immunocytochemistry and qPCR.

**Results:**

In contrast to miR-140, the expression of *WWP2* was similar in both normal and OA cells, suggesting that miR-140 has an additional level of regulation. rsmiR-140 showed activity and predicted binding sites for nuclear matrix transcription factor 4 (*NMP4*), myc-associated zinc (*MAZ*), nuclear factor of activated T-cells (*NFAT*), and mothers against decapentaplegic homolog 3 (*SMAD3*). Silencing *NFAT3* (*P* ≤0.01) and *SMAD3* (*P* ≤0.05) differentially regulated miR-140 independently of *WWP2*. Silencing *NFAT5* decreased both miR-140 and *WWP2* (*P* ≤0.003 and *P* ≤0.05, respectively). *NFAT3* activation increased and transforming growth factor-β (TGF-β) decreased rsmiR-140 activity. Mutagenesis of rsmiR-140 and ChIP assays identified binding sites at which NFAT3 (activator) and SMAD3 (repressor) directly regulated miR-140. TGF-β interfered with NFAT3 translocation, and subsequently with miR-140 expression.

**Conclusions:**

This is the first study to provide evidence of a regulatory mechanism of miR-140 independent of *WWP2*, and new and differential roles for NFAT3 and SMAD3 in the OA process in the regulation of miR-140 transcription. Such knowledge could advance therapeutic strategies targeting OA.

## Introduction

MicroRNAs (miRNAs) add another level of regulation to gene expression by down-regulating their target genes. Some miRNAs including miR-146 and miR-155 have been linked to arthritis pathologies, such as rheumatoid arthritis [1-3], but miR-140, originally found in cartilage [4], has been linked more specifically to osteoarthritis (OA) [5,6]. miR-140 decreases the expression of genes known to play detrimental roles in OA cartilage. Among them are histone deacetylase 4 [4], which was recently shown to interact with Runx2, a repressor of matrix metalloproteinase-13 (*MMP-13*) transcription [7], A disintegrin and metalloproteinase with a thrombospondin type 1 motif (*ADAMTS-5*) [8], whose deletion generated OA-like changes [9,10], mothers against decapentaplegic homolog 3 (*SMAD3*) [11], a mediator of transforming growth factor-β (TGF-β) signaling reported to be associated with hip and knee OA in European populations [12] and insulin-like growth factor-binding protein-5 (*IGFBP5*) [5] an important factor in IGF-1 storage in the joint [13] whose increase is associated with reduced cartilage destruction [14]. Targeted deletion of miR-140 in mice resulted in age-related OA-like changes [8]. Of importance, miR-140 expression is significantly decreased in human OA chondrocytes [5,6], thus favouring an increased expression of its target genes and consequently a role in cartilage degradation.

miR-140 is found in one intron of the WW domain containing E3 ubiquitin protein ligase 2 (*WWP2*) gene [15]. Analysis of the intronic sequence has revealed the presence of two miR-140 s, miR-140-5p and miR-140-3p. All of the previous studies done with arthritic cells and tissues used miR-140-5p. Although both miR-140-5p and -3p are transcribed from the same precursor transcript pre-miR-140, they have different seed sequences and are, therefore, predicted to target different genes. While miR-140-5p was shown to target several genes involved in OA, miR-140-3p has been reported to target dynamin 1, which plays a role in the central nervous system [16] and the nuclear factor kappa B (NF-κB) co-activator nuclear receptor-interacting protein 1 [17].

Because of its role in inhibiting key factors involved in OA pathophysiology and its down-regulation in OA cartilage, understanding the transcriptional regulation of miR-140 in this pathological condition is of great importance and could open up new therapeutic avenues targeting this disease.

## Methods

### Specimen selection

Human cartilage was obtained from femoral condyles and tibial plateaus. Normal human cartilage was obtained from individuals within 12 hours of death (n = 8, 60 ± 19 years (mean ± SD)) and OA cartilage from patients undergoing total knee arthroplasty (n = 48, 66 ± 9 years). Normal individuals had no history of joint disease and died of causes unrelated to arthritic diseases. The cartilage was examined macroscopically and microscopically to ensure that only normal tissue was used. All OA patients had been evaluated by a certified rheumatologist and diagnosed as having OA according to the American College of Rheumatology (ACR) criteria [18]. These specimens represented moderate to severe OA. The Institutional Ethics Committee Board of the University of Montreal Hospital Research Centre (CRCHUM) approved the use of the human articular tissues. Patients signed informed consent and post-mortem tissue was obtained with the consent of a family member or authorized individual.

### Cell culture

Chondrocytes were seeded directly from the digested cartilage (primary chondrocytes) as described [5] and the SW1353 chondrosarcoma cell line was purchased from American Type Culture Collection, Manassas, VA, USA (#HTB-94). Briefly, the cells were seeded at high density (10^5^/cm^2^) and cultured in (Dulbecco’s) modified Eagle’s medium ((D)MEM; Wisent, St-Bruno, QC, Canada) supplemented with 10% heat-inactivated fetal calf serum (FCS; PAA Laboratories Inc, Etobicoke, ON, Canada) and an antibiotic mixture (100 units/ml penicillin base and 100 μg/ml streptomycin base; Wisent) at 37°C in a humidified atmosphere. Primary chondrocytes were used when comparing expression levels in normal and OA chondrocytes to avoid loss of the chondrocyte phenotype and first-passage chondrocytes for all other experiments involving cultured chondrocytes. In the experiments, the culture medium was replaced with (D)MEM containing 0.5% FCS 24 hours before the treatment. The ionophore ionomycin (1 μM; Sigma, Oakville, ON, Canada), NaCl (100 mM), and TGF-β (10 ng/ml, Feldan Bio Inc., Montreal, QC, Canada) were added for 18 hours and the specific inhibitor of SMAD3 phosphorylation (SIS3, 8 μM; Calbiochem, La Jolla, CA, USA) [19] for 24 hours.

### Quantification of mRNA and miRNA

Total RNA was extracted and quantified as described [5] except that 10 μg glycogen was added to the precipitation step to enrich for miRNAs. mRNA levels were quantified by real-time PCR with the SYBR Green PCR Master Mix (Qiagen, Valencia, CA, USA). When quantifying expression between normal and OA chondrocytes, internal standards were added at known concentrations in the PCR reactions and amplified by the same primers as the specific target mRNAs as to give absolute numbers. The values of each sample were calculated as the ratio of the number of molecules of the target gene/number of molecules of the housekeeping gene. When evaluating the effect of a treatment on a given cell culture, the expression level of each control was assigned an arbitrary value of 1, and the treated cells were evaluated as fold change over control and calculated as 2^-Δ(ΔCt)^. Basal expression values of the control specimens (as determined from the number of molecules of the target gene/housekeeping gene) are shown in Additional file 1: Figure S1. Primer efficiencies for the genes under study were the same as those for the housekeeping gene glyceraldehyde 3-phosphate dehydrogenase (*GAPDH*). The sequences of the human specific primers used are listed in Table 1. miRNAs were quantified with the TaqMan MicroRNA Reverse Transcription kit and TaqMan MicroRNA Assays specific for each mature miRNA (Applied Biosystems, Burlington, ON, Canada) as described [5]. Normalisation of the miRNA expression data was done using the *GAPDH* gene. The expression level of each control was assigned an arbitrary value of 1 and the treated cells were evaluated as fold change over control.

**Table 1:** Sequences of the human gene-specific primers used for qPCR

**Gene**	**Sequence**
**(GenBank accession number)**	
FAK	5’-AGAAGTATGAGCTTGCTCAC (S)
(NM_153831.2)	5’-TGATCGCCGTATTTCTAGAC (AS)
GAPDH	5’-CAGAACATCATCCCTGCCTCT (S)
(NM_002046.3)	5’-GCTTGACAAAGTGGTCGTTGAG (AS)
IGFBP5	5’-TGAAGCAGTGAAGAAGGAC (S)
(NM_000599)	5’-CTGCTTTCTCTTGTAGAATC (AS)
MAZ	5’-GATCACCTCAACAGTCACGTC (S)
(NM_002383.2)	5’-CTGTGCACCTTCATGTGGTC (AS)
NFAT1 (NFATc2)	5’-AGAATCCATCCTGCTGGTTC (S)
(NM_012340)	5’-TCCATGTAGCCATGGAGCTG (AS)
NFAT2 (NFATc1)	5’-TCATTGACTGTGCCGGAATC (S)
(NM_172390)	5’-AAGTTGTGGCCAGACAGGAC (AS)
NFAT3 (NFATc4)	5’-AGAACTGGACTCAGAGGATG (S)
(NM_004554)	5’-ATGGAGGTGATGCGGATG (AS)
NFAT4 (NFATc3)	5’-CCAGCCCGGGAGACTTCAATAGAT (S)
(NM_173165)	5’-GCCCAGGAGCTTCACAACAGGAT (AS)
NFAT5 (TonEBP)	5’-CTGAGCAGAGCTGCAGTATG (S)
(NM_138713)	5’-GTTGTCCGTGGTAAGCTGAG (AS)
NMP4	5’-CGTACTTCTGGCCTTCTATC (S)
(AB070238)	5’-CAGTCATCAGTCCTGTAGAC (AS)
SMAD3	5’-GTCTGCAAGATCCCACCAGG (S)
(NM_005902)	5’-CTTGTCAAGCCACTGCAAAG (AS)
SREBF2	5’-TGAGATCCATCTGACTGCTG (S)
(NM_004599)	5’-CCTCTGGGCACAGTATAGAC (AS)
WWP2	5’-CAAGGTGCATAATCGTCAAC (S)
(NC_000016) (variants 1, 3)	5’-GATGCGGTGCCTAGCAGTTC (AS)
WWP2	5’-TCCTCCTGTCTCATGAGGTG (S)
(NC_000016) (variants 1, 2)	5’-GCCTATAAAGCGAAAGTAGG (AS)

*S* sense, *AS* antisense.

### Gene silencing

siRNA pools specific for *NMP4, MAZ, NFAT1* (NFATc2), *NFAT2* (NFATc1), *NFAT3* (NFATc4), *NFAT4* (NFATc3), *NFAT5* (TonEBP) and *SMAD3* were purchased from Dharmacon (Lafayette, CO, USA) and transfected with the HiPerfect Transfection Reagent (Qiagen) at a concentration of 100 nM. Total RNA was extracted after 48 hours and mRNA levels measured by quantitative PCR and normalised to the control gene. Cells transfected with non-targeting (random) siRNAs served as controls. Silencing gene expression was carried out by transfecting OA chondrocytes for 48 hours with small interfering RNAs (siRNAs), resulting in a silencing efficiency of more than 80% as described [20] and as illustrated in Additional file 2: Figure S2.

### Cloning of the 5’-flanking region of pre-miR-140 (rsmiR-140)

1.883 kb of the 5’-flanking region directly upstream of pre-miR-140 (regulatory sequence miR-140 (rsmiR-140)) was cloned into the luciferase reporter vector pGL3-basic (Promega, Madison, WI, USA). The fragment was amplified by PCR using genomic DNA from human chondrocytes and the primers 5’-TGA**GCTAGC**GGTGCTTATGACCGCAGTTTTC (sense) and 5’-CAG**AAGCTT**ACCAAGCAGAGCCTGGAGAGGAG (antisense). NheI and HindIII restriction sites (bold) were added to the sense and antisense primers, respectively, to facilitate the cloning into the NheI and HindIII sites of the pGL3-basic vector. A smaller plasmid was constructed similarly by using the sense oligonucleotide 5’-TGA**GCTAGC**GTGCCCCGGAAGGCTGCCCTGTAC, resulting in a cloned fragment of 1.153 kb. Both plasmids were sequenced to confirm the integrity of the cloned DNA.

Wild-type and mutated oligonucleotides were chemically synthesised and used in site-directed mutagenesis assays with the QuickChange Site-Directed Mutagenesis kit (Stratagene, La Jolla, CA, USA) as described [21]. Bases were mutated to change the consensus NFAT binding site GGAAA to GGTAC, and the SMAD3 consensus binding sites from CAGA to TACC and from TTGGTGTTGG to TTGCATATGG. Each plasmid was verified by DNA sequencing.

### Transfection and rsmiR-140 activity

The SW1353 cells were transfected with the TransFectin transfection reagent (Bio-Rad, Mississauga, ON, Canada), incubated for 48 hours in (D)MEM/10% FCS and lysed in Reporter Lysis buffer (Promega). The transfected cells were then incubated for an additional 18 hours with the factor under study. Luciferase activity was measured with the Lumat LB 9507 luminometer (EG&G Berthold, Bad Wildbad, Germany). To monitor transfection efficiency, a plasmid coding for a green fluorescent protein was used. Total protein was quantified by the bicinchoninic acid method (Pierce, Rockford, IL, USA). Plasmid activity was calculated as relative luciferase units per μg of protein. The pGL3-basic control was assigned an arbitrary value of 1 for each experiment and the activity evaluated as fold change over control. Basal expression values of the control specimens as determined by luciferase units/μg of protein are shown in Additional file 1: Figure S1.

### Western blotting

OA chondrocytes were treated with ionomycin (1 μM, 60 minutes), NaCl (100 mM, 60 minutes) or TGF-β (10 ng/ml, 30 minutes). Nuclear proteins from the control and treated cells were extracted with the Nuclear Extraction kit (Cayman Chemical Company, Ann Arbor, MI, USA) and processed (5 μg) for Western blotting as previously described [21]. The primary antibodies were a rabbit anti-human SMAD3 (Cell Signaling Technology, Danvers, MA, USA; dilution 1/2,000), a mouse anti-human NFAT3 (Santa Cruz Biotechnology, Santa Cruz, CA, USA; dilution 1/2,000), and a rabbit anti-NFAT5 (Novus Biologicals, Littleton, CO, USA; dilution 1/5,000). The secondary antibodies were an anti-mouse or anti-rabbit immunoglobulin G (IgG; Pierce, Rockford, IL, USA; dilution 1/10,000). The nuclear protein nucleolin (Active Motif, Carlsbad, CA, USA; dilution 1/5000) was used as a housekeeping (control) protein.

### Chromatin immunoprecipitation

OA chondrocytes were treated with TGF-β (10 ng/ml, 30 minutes) or ionomycin (1 μM, 60 minutes) and processed with the EZ-Magna ChIP A/G Chromatin Immunoprecipitation Assay kit (Millipore, Temecula, CA, USA) as recommended by the manufacturer and as described [22]. The antibodies used in the chromatin immunoprecipitation (ChIP) reactions were an anti-human SMAD3 (Cell Signaling Technology) and an anti-human NFAT3 (Santa Cruz Biotechnology). Pre-immune IgGs were used as negative control. The PCR primers were 5’-GCATTGTCTTGCCTTCACCC (sense, located 52 bp upstream of the NFAT3 binding site) and 5’-TGGAGAGGAGGTCACCACGG (antisense, located 200 bp downstream from the NFAT3 site). These primers were also used for the SMAD3 ChIP assay, as the NFAT3 and SMAD3 sites are separated by less than 40 bp. The primer sequences of the *MAP1A* gene (microtubule-associated protein 1A, used as an unrelated control gene) were 5’-GACCTTATGCCAGGACAGGA (sense) and 5’-AGACAAGAGCTCCCTCCACA (antisense). The pre-immune IgG ChIP results were used as negative control and the data from the NFAT3 and SMAD3 experiments were normalized to this control. The genomic DNA (1% of the starting lysate input) was used as positive control. The amplified PCR products were analysed on agarose gels and quantitated by qPCR. The results were compared to those of the pre-immune assays, and the effect of the treatment measured as fold change over the control which was assigned an arbitrary value of 1. Basal expression values of the control specimens (as determined by PCR control values per input control DNA) are shown in Additional file 1: Figure S1.

### Nuclear translocation

Nuclear translocation of SMAD3 and NFAT3 was assessed in OA chondrocytes by immunocytochemistry and its effect by qPCR.

#### Immunocytochemistry

Cells were cultured on Permanox slides (Thermo Fisher Scientific, Ottawa, ON, Canada). The effect of TGF-β on NFAT3 translocation was monitored by treating the cells with ionomycin (1 μM) for 90 minutes, with TGF-β (10 ng/ml) added for the last 30 minutes of the incubation. The effect of ionomycin/NFAT3 on SMAD3 translocation was examined by treating the cells with TGF-β (10 ng/ml) for 90 minutes and ionomycin (1 μM) for the last 60 minutes. The cells were fixed with 4% paraformaldehyde for 30 minutes at 4°C, washed with PBS and treated with 10% NH_4_Cl. The cells were then permeabilised with 0.3% Triton X-100 for 30 minutes at room temperature, blocked with 1% BSA for one hour at room temperature, and probed with the specific antibodies overnight at 4°C.

The primary antibodies were a rabbit anti-human SMAD3 (1 μg/ml, Abcam, Cambridge, MA, USA) and a mouse anti-human NFAT3 (5 μg/ml, Santa Cruz Biotechnology). The slides were then incubated for one hour at room temperature with the secondary antibodies (10 μg/ml, biotinylated anti-mouse or anti-rabbit IgG, Vector Laboratories, Burlingame, CA, USA and Life Technologies, Burlington, ON, Canada, respectively). Streptavidin-coupled ALEXA 488 (10 μg/ml, Life Technologies) was added (one hour in the dark at room temperature) and the nuclei were counterstained with 4’,6-diamidino-2-phenylindole, dihydrochloride (DAPI, Molecular Probes, Eugene, OR, USA). The slides were mounted with Vectashield (Vector Laboratories). Images were acquired using a Zeiss Axio microscope and its processing software AxioVision with the monochromatic AxioCam MRm camera (Zeiss Canada, Toronto, ON, Canada).

Control procedures were performed according to the same experimental protocol by (i) omission of the primary antibody, and (ii) substitution of the primary antibody with a non-specific IgG from the same host as the primary antibody (Santa Cruz Biotechnology). Control slides showed only background staining. The total number of chondrocytes and the number of chondrocyte nuclei staining positive for NFAT3 or SMAD3 were counted in five random representative fields. Results were calculated as the percentage of the total number of chondrocytes staining positive.

#### Effect of nuclear translocation on miR-140 expression

OA chondrocytes were incubated with ionomycin (1 μM) or TGF-β (10 ng/ml) for two to twenty hours; TGF-β was added during the ionomycin treatment and ionomycin added during the TGF-β treatment. Preliminary experiments revealed a maximum effect at eight hours, with TGF-β added to the last 6½ hours of the ionomycin treatment and vice-versa. miR-140 expression was determined by qPCR as above.

### Statistical analysis

Values are expressed as mean ± standard error of the mean (SEM). Statistical significance was assessed with the Mann–Whitney test or a one-sample t-test where appropriate; a *P* value ≤0.05 was considered significant.

## Results

### Differential expression levels of miR-140 and *WWP2*

**Figure 1:**
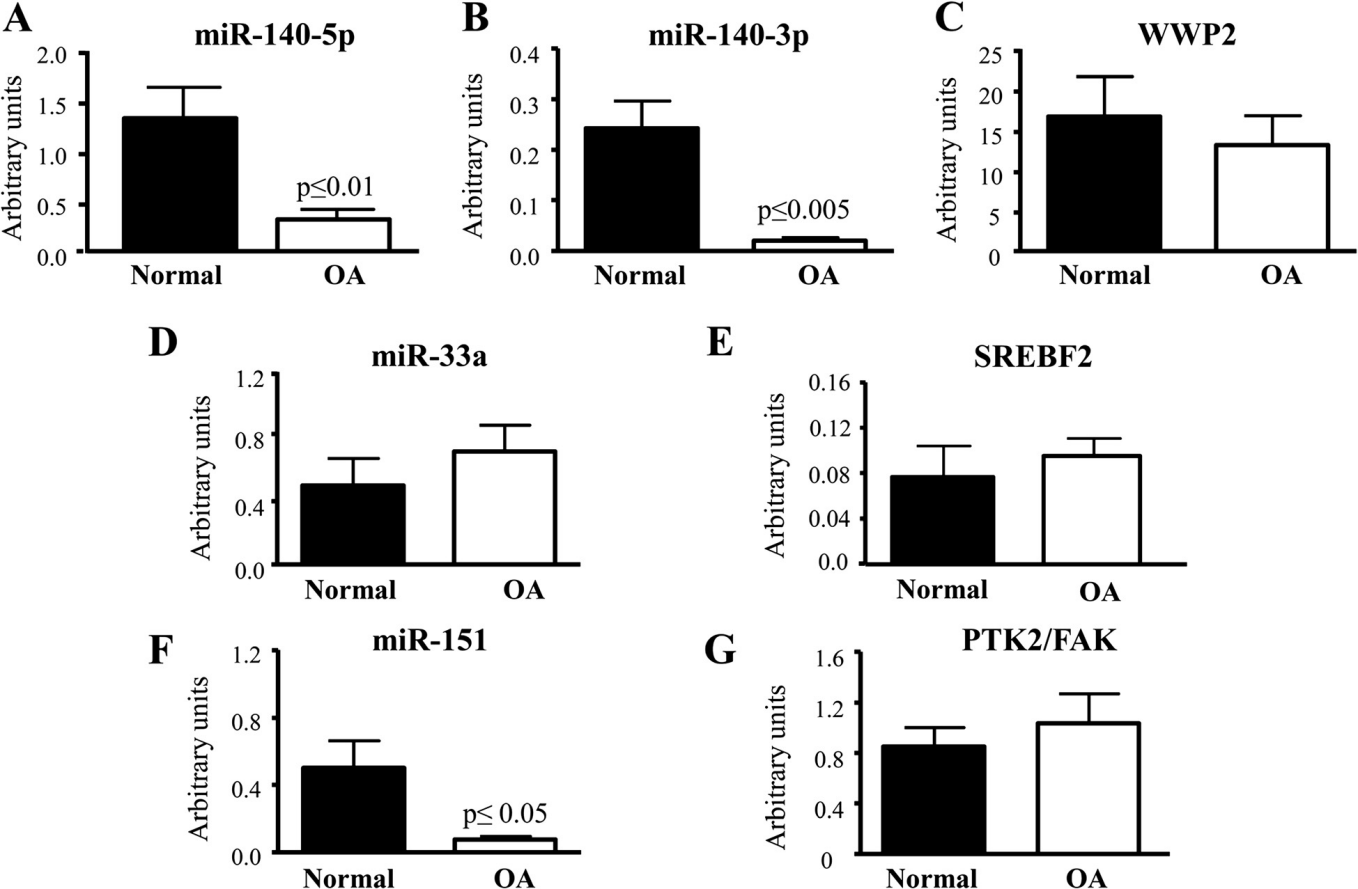
**Expression of miRNAs and their host genes.** Expression (number of molecules of the target gene/number of molecules of the housekeeping gene) of the intronic miRNAs **A)** miR-140-5p, **B)** miR-140-3p, and **C)** their host gene WWP2, of **D)** miR-33a and **E)** its host gene SREBF2, of **F)** miR-151 and **G)** its host gene PTK2/FAK in human normal (n = 6) and osteoarthritic (OA) (n = 6) chondrocytes. *P* values were assessed by the Mann–Whitney test comparing OA to normal chondrocytes. miRNAs, microRNAs.

We previously reported that miR-140-5p expression was significantly reduced in human OA chondrocytes [5]. Here, we followed this by comparing its expression to that of miR-140-3p, and their host gene, *WWP2*, in normal and OA human chondrocytes. Data showed (Figure 1A, B) that the expression levels of miR-140-5p and -3p were both markedly and significantly reduced in OA chondrocytes. Interestingly, *WWP2* expression (Figure 1C) was only slightly reduced (about 21%) in OA and this did not reach statistical significance. Of note, these results represent the global expression of the mRNAs and miRNAs at a given point in time.

The *WWP2* gene codes for three variants or isoforms. Variant 1 (FL-isoform) is the longest variant with 25 exons; variant 2 (C-isoform) has its ATG in exon 14 of variant 1 and the same TAA stop codon as variant 1; variant 3 (N-isoform) has the same ATG as variant 1 but with a stop codon in exon 10. Preliminary RT-PCR experiments using primers specific for variants 2 and 3 [23] have shown that these variants were not as strongly expressed as variant 1 in human chondrocytes. Variant 3 was expressed in both normal and OA chondrocytes and the expression pattern was similar to that observed with variant 1 (data not shown); variant 2 was either not expressed or very weakly expressed in chondrocytes (data not shown). We have routinely used primers located in exons 4 and 5 (variants 1 and 3, Figure 1C). Experiments performed with primers located in exons 14 and 16 (variants 1 and 2) (Additional file 3: Figure S3 A) showed similar data to those with the variants 1 and 3 and yielded no significant differences between the two sets of primers. Subsequent experiments were done using primers located in exons 4 and 5.

To determine whether the differential expression level between *WWP2* and miR-140 was due to a different miRNA processing in OA cells, we determined the levels of two unrelated intronic miRNAs: miR-33a (Figure 1D) present in one intron of the sterol regulatory element binding factor-2 (*SREBF2*) gene and miR-151 (Figure 1F), present in one intron of the protein tyrosine kinase or focal adhesion kinase (*PTK2/FAK*) gene. The expression levels of miR-33a (Figure 1D) and its host gene *SREBF2* (Figure 1E) were similar in normal and OA chondrocytes; however, the expression level of miR-151 (Figure 1F) was significantly decreased compared to that of its host gene *PTK2/FAK* (Figure 1G). These findings indicate that the reduced expression of miR-140 in OA chondrocytes is not due to a general processing and likely results from an additional level of regulation specifically directed at this miRNA.

### Identification of an intronic regulatory sequence upstream of pre-miR-140

**Figure 2:**
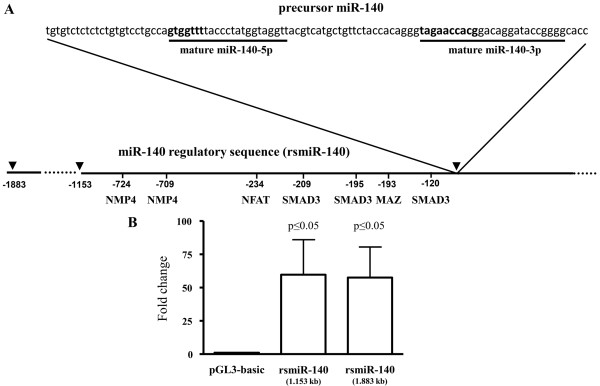
**miR-140 sequence and activity of rsmiR-140. A)** Sequence of the miR-140 precursor (pre-miR-140) stem-loop located in intron 16 of the WWP2 gene. The letters in bold represent the seed regions of the mature miRNAs. The arrows delineate the DNA sequence upstream of the pre-miR-140 (shown between the diagonal lines) that was cloned into the pGL3-basic vector and identified as miR-140 regulatory sequence (rsmiR-140) with the location of potential transcription factor binding sites. **B)** The rsmiR-140 (1.153 kb and 1.883 kb) activities were determined in SW1353 cells (n = 4). Plasmid activity was calculated as relative luciferase units per μg of protein; the control (pGL3-basic) was assigned an arbitrary value of 1 for each experiment and the rsmiR-140 activity evaluated as fold change over control. *P* values were assessed by the one-sample t-test, comparing rsmiR-140 to the pGL3-basic vector.

Intronic miRNAs can be regulated independently of their host gene by sequences located directly upstream of their precursor sequence. To determine if such was the case for miR-140 and *WWP2*, we cloned 1.883 kb and 1.153 kb (Figure 2A) of the sequence located directly upstream of the pre-miR-140 [GenBank:NC_000016]. Both plasmids promoted similar transcriptional activity (Figure 2B), indicating the presence of regulatory elements in the sequence upstream of pre-miR-140. Further experiments were conducted with the 1.153 kb cloned DNA, designated in the text rsmiR-140.

### Identification of NFAT3 and SMAD3 as regulators of miR-140 expression independent of *WWP2*

**Figure 3:**
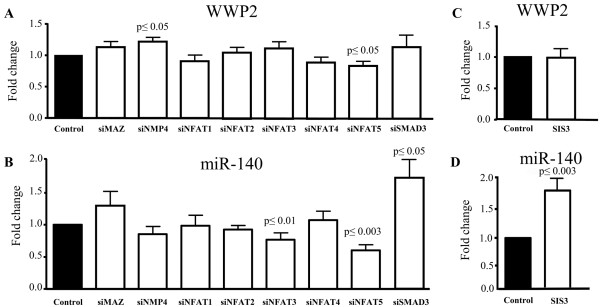
**Inhibition of expression (siRNAs) and phosphorylation (SIS3).** Effect of silencing (siRNA) the expression of MAZ, NMP4, NFAT1-5, and SMAD3 on **A)** WWP2 and **B)** miR-140 expression in human osteoarthritic chondrocytes (n = 7 to 10). Effect of inhibiting SMAD3 phosphorylation following incubation of osteoarthritic chondrocytes (n = 8) without (control) or with the specific SMAD3 phosphorylation inhibitor SIS3 on the **C)** WWP2 and **D)** miR-140 expression. Each control (random-treated cells) was assigned an arbitrary value of 1 and the effect of the treatment evaluated as fold change over control. *P* values were assessed by the one-sample t-test, comparing the treated chondrocytes to the controls.

The rsmiR-140 sequence has several potential binding sites for transcription factors, such as NMP4 (CAAAAA), MAZ (GAGAGA), NFAT (GGAAA) and SMAD3 (CAGA, TTGGTGTTGG) (Figure 2A). To examine whether these factors could be responsible for the differential regulation of miR-140 and *WWP2*, their expression was silenced in OA chondrocytes and the miR-140 and *WWP2* levels were determined (Figure 3). As both miR-140 s are similarly decreased in OA chondrocytes, further experiments were carried out with miR-140-5p.

Silencing *NFAT3* significantly decreased (*P* ≤0.01) miR-140 expression without affecting *WWP2*, and silencing *SMAD3* significantly increased (*P* ≤0.05) miR-140 without significantly affecting *WWP2*. Silencing *NFAT5* significantly decreased (*P* ≤0.003) miR-140 and, to a lesser extent, *WWP2* expression (*P* ≤0.05). Silencing *NMP4* significantly increased *WWP2* (*P* ≤0.05) but not miR-140, and silencing *NFAT1, NFAT2, NFAT4* or *MAZ* did not significantly affect either miR-140 or *WWP2* levels. To further investigate SMAD3, OA chondrocytes were treated with the specific inhibitor of SMAD3 phosphorylation SIS3 (Figure 3C, D). Data showed a pattern similar to that of the silenced *SMAD3;WWP2* expression was not affected (Figure 3C) and miR-140 expression was significantly increased (*P* ≤0.003) (Figure 3D).

**Figure 4:**
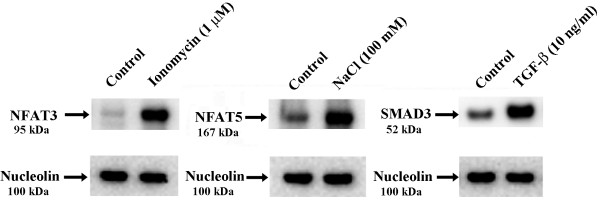
**Nuclear translocation of NFAT3, NFAT5 and SMAD3 as determined by Western blotting.** Human osteoarthritic chondrocytes (n = 4) were treated with ionomycin (60 minutes), NaCl (60 minutes) and TGF-β (30 minutes) to activate and translocate NFAT3, NFAT5 and SMAD3, respectively. Nuclear extracts from the control and treated cells were processed for Western blotting with specific antibodies. Nucleolin, a housekeeping nuclear protein, was used as control. TGF-β, transforming growth factor β.

**Figure 5:**
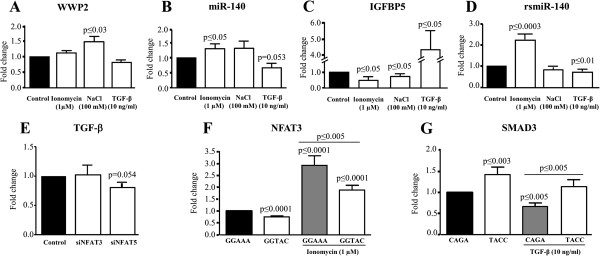
**Effect of ionomycin, NaCl, and TGF-β on miRNA and mRNA expression and rsmiR-140 activity.** The effect of increasing calcium flux (ionomycin), hypertonic stress (NaCl) and TGF-β on **A)** WWP2, **B)** miR-140 and **C)** IGFBP5 expression in human osteoarthritic chondrocytes (n = 7 to 12), and on **D)** the miR-140 regulatory sequence (rsmiR-140) in SW1353 cells (n = 6 to 9). **E)** Effect of silencing NFAT3 and NFAT5 on TGF-β expression in human osteoarthritic chondrocytes (n = 5). Effect of specific mutations of **F)** NFAT3 (−234 bp GGAAA to GGTAC) and **G)** SMAD3 (−195 bp CAGA to TACC) binding sites on rsmiR-140 activity in SW1353 (n = 10). Each control (untreated cells) was assigned an arbitrary value of 1 and the effect of the treatment evaluated as fold change over control. *P* values were assessed by the one-sample t-test, comparing treated chondrocytes to the controls or as underlined. miRNA, microRNA; TGF-β, transforming growth factor β.

The effects of SMAD3, NFAT3 and NFAT5 were investigated in OA chondrocytes by activating SMAD3 by TGF-β, NFAT3 by increasing calcium flux with ionomycin and NFAT5 by hypertonic stress via increasing NaCl concentration. The concentrations used and durations of exposure resulted in an accumulation of the molecules in the nucleus as shown by Western blot (Figure 4). As illustrated in Figure 5A and B, ionomycin significantly increased (*P* ≤0.05) miR-140 but not *WWP2* expression, while NaCl increased both *WWP2* and miR-140 expression levels but significance was reached only for *WWP2* (*P* ≤0.03). TGF-β significantly decreased (*P* ≤0.05) miR-140 but had no true effect on *WWP2* levels. Complementary experiments evaluating the expression of the *WWP2* variants 1 and 2 also showed no effect of ionomycin or TGF-β, but a significant increase with NaCl treatment (Additional file 3: Figure S3 B). Additional experiments were performed to determine the expression level of a known miR-140 direct target, *IGFBP5*. Data demonstrated (Figure 5C) that the increased expression of miR-140 following stimulation by ionomycin and NaCl resulted in decreased expression of *IGFBP5*, and the TGF-β-induced decrease in miR-140 led to an increased expression of *IGFBP5*, indicating that a change in miR-140 expression was reflected on its target gene.

All together, these findings confirm that NFAT3 and SMAD3 affect miR-140 expression independently of *WWP2*. It is possible that the increased expression of miR-140 caused by NFAT5 under hypertonic conditions results in part from increased expression of its host gene *WWP2*.

### Ionomycin and TGF-β regulate rsmiR-140 activity

We further examined whether the regulation by NFAT3, NFAT5 and SMAD3 of miR-140 occurred at the level of rsmiR-140. SW1353 cells were transfected with the rsmiR-140 plasmid and treated with ionomycin, NaCl and TGF-β (Figure 5D). rsmiR-140 was significantly stimulated by ionomycin (*P* <0.0003), decreased by TGF-β (*P* <0.01) and not affected by NaCl. To verify that SMAD3, but not SMAD1, was involved in miR-140 regulation, the cells were treated with BMP2 (10 ng/ml); rsmiR-140 activity was not affected by this factor (data not shown). This result agrees with our previous finding that BMP2, as opposed to TGF-β, does not significantly affect miR-140 expression [5].

We next investigated whether NFAT3 and NFAT5 could act through TGF-β, as the TGF-β promoter contains potential NFAT binding sites [GenBank:J04431.1]. *NFAT3* and *NFAT5* expression was silenced in OA chondrocytes and the TGF-β levels determined (Figure 5E). Interestingly, NFAT3 did not affect *TGF-β* expression, but NFAT5 significantly decreased its levels (*P* = 0.054).

Together, these results indicate that the TGF-β-induced miR-140 down-regulation is the result of SMAD3 activation and that NFAT3 regulates miR-140 directly, likely at the rsmiR-140 level. NFAT5 could indirectly contribute to the down-regulation of miR-140 by up-regulating the expression of TGF-β, which in turn inhibits miR-140 expression.

### Identification of NFAT and SMAD3 regulatory binding sites on rsmiR-140

rsmiR-140 has consensus binding sites for NFAT (GGAAA at position -234 bp) and SMAD3 (CAGA at position -209 bp and -195 bp, and TTGGTGTTGG at -120 bp) (Figure 2A). To determine if NFAT3 and SMAD3 directly acted through those sites, SW1353 cells were transfected with rsmiR-140 without or with the mutated sites and treated with ionomycin and TGF-β. Mutation of the NFAT site (Figure 5F) significantly decreased basal (*P* ≤0.0001) as well as the ionomycin-induced (*P* ≤0.0001) expression, indicating the involvement of this site in the positive regulation of miR-140 by NFAT3. The -195 bp CAGA mutation (Figure 5G) resulted in a significant increase in basal (*P* ≤0.003) and TGF-β-induced (*P* ≤0.005) expression, suggesting its involvement in the negative regulation of miR-140 by TGF-β through the inhibitory action of SMAD3. Mutating the -120 bp TTGGTGTTGG and -209 bp CAGA sites did not affect either basal or TGF-β-induced expression (data not shown). It was also observed that TGF-β treatment of the -195 bp mutated construct resulted in a slightly decreased expression. TGF-β has a number of roles and acts through many intermediates in addition to SMAD3; it is thus possible that TGF-β could act indirectly on rsmiR-140 through other (unknown) binding sites.

### NFAT3 and SMAD3 physically interact with rsmiR-140

**Figure 6:**
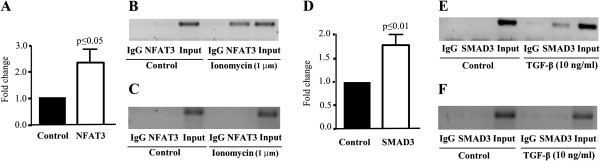
**ChIP assays of NFAT3 and SMAD3 on rsmiR-140.**. Human osteoarthritic chondrocytes (n = 7 to 8) in **A)** NFAT3 chromatin immunoprecipitation (ChIP) assay and representative gels using primers specific to the **B)** rsmiR-140 sequence (amplified fragment of 253 bp) and **C)** unrelated control gene MAP1A (amplified fragment of 211 bp); **D)** SMAD3 ChIP assay and representative gels using primers specific to the **E)** rsmiR-140 sequence (amplified fragment of 253 bp) and **F)** unrelated control gene MAP1A (amplified fragment of 211 bp). IgG: pre-immune IgG, input: 1% of the starting genomic DNA lysate. Cells received no treatment (control) or were treated with **B)**, **C)** ionomycin or **E)**, **F)** TGF-β. Each control (panels **A** and **D**) was assigned an arbitrary value of 1 and the amplification evaluated as fold change over control. *P* values were assessed by the one-sample t-test, comparing treated chondrocytes to controls. IgG, immunoglobulin G; TGF-β, transforming growth factor β.

ChIP assays were done to determine if NFAT3 and SMAD3 physically associated with the identified sites. OA chondrocytes were treated with ionomycin and TGF-β and processed for ChIP assays (Figure 6). The results (Figure 6A, B, D, E) showed that treatment with ionomycin and TGF-β significantly enriched the DNA sequences containing the binding sites of NFAT3 and SMAD3 (*P* ≤0.05, *P* ≤0.01, respectively). Similar experiments done with primer pairs located 800 bp upstream of the binding sites and on the unrelated negative control gene *MAP1A* revealed no significant binding (Figure 6C, F), suggesting that the increased binding seen with treatment with TGF-β and ionomycin was specific for rsmiR-140.

### TGF-β interferes with NFAT3 translocation

**Figure 7:**
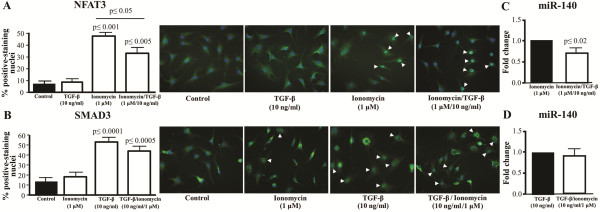
**Translocation of NFAT3 and SMAD3 to the nucleus.** Translocation of **A)** NFAT3 and **B)** SMAD3 in the nuclei of human osteoarthritic chondrocytes (n = 7 to 10). For NFAT3, cells received no treatment (control) or were treated with TGF-β for 30 minutes or with ionomycin for 90 minutes without or with TGF-β added for the last 30 minutes. For SMAD3, cells received no treatment (control) or were treated with ionomycin for 60 minutes or with TGF-β for 90 minutes without or with ionomycin added for the last 60 minutes. Representative immunocytochemistry of the translocation of **A)** NFAT3 in the control, TGF-β, ionomycin, and ionomycin/TGF-β treated osteoarthritic chondrocytes for the abovementioned time period, and **B)** SMAD3 in the control, ionomycin, TGF-β, and TGF-β/ionomycin-treated cells for the abovementioned time period. The percentage of NFAT3 and SMAD3 (green stain) nuclear translocation was calculated relative to the number of total cells (nuclei stained blue with DAPI). The white arrow-heads identify positive-nuclei. P values were assessed by the Mann–Whitney test comparing treatments to controls or as underlined. Effect of **C)** TGF-β on the ionomycin-induced expression of miR-140 and of **D)** ionomycin on the TGF-β-induced expression of miR-140. Human osteoarthritic chondrocytes (n = 7) were treated for 8 hours with ionomycin with or without the addition of TGF-β for the last 6 1/2 hours, or with TGF-β for 8 hours without or with the addition of ionomycin for the last 6 1/2 hours. RNA was extracted and the expression of miR-140 was measured. **C)** and **D)** each control (ionomycin and TGF- β, respectively) was assigned an arbitrary value of 1 and the effect of the double treatment evaluated as fold change over control. *P* values were assessed by the one-sample t-test, comparing the double treated chondrocytes to the autologous control. DAPI, 4’,6-diamidino-2-phenylindole, dihydrochloride; TGF-β, transforming growth factor β.

As TGF-β production is significantly increased in OA [24,25], we examined whether TGF-β could interfere with the translocation of NFAT3 and prevent its action. As expected (Figure 7A, B), ionomycin significantly triggered the translocation of NFAT3 (47%, *P* ≤0.001), and TGF-β triggered that of SMAD3 (52%, *P* ≤0.0001) in OA chondrocytes. Treatment with TGF-β alone had no significant effect on NFAT3 translocation and treatment with ionomycin alone had no effect on SMAD3 translocation. However, when TGF-β was added for the last 30 minutes of the 90-minute ionomycin treatment, there was a significant decrease in the number of NFAT3-positive nuclei (*P* ≤0.05) when compared to ionomycin alone (Figure 7A). When ionomycin was added for the last 60 minutes of the 90-minute TGF-β treatment, there was a slight but non-significant decrease in SMAD3-positive nuclei (Figure 7B). Similar experiments done with human normal chondrocytes revealed the same pattern, that is, TGF-β interfered with the ionomycin-induced translocation of NFAT3 into the nucleus (data not shown).

Further experiments were done to verify whether such interference affected miR-140 expression (Figure 7C, D). The addition of TGF-β during the ionomycin treatment resulted in a significant decrease (*P* ≤0.02) in miR-140 compared to ionomycin treatment alone, while the addition of ionomycin to the TGF-β treatment did not significantly affect the miR-140 expression level. All together, these results indicate that the presence of TGF-β interferes with the ionomycin-induced nuclear translocation of NFAT3 and ultimately miR-140 expression.

## Discussion

Our previous study in human chondrocytes [5] and those of others in mouse cells [6,8] identified target genes down-regulated by miR-140 that are important in the OA cartilage process.

We report, for the first time, a differential expression between miR-140 and its host gene. We also identify a regulatory sequence located directly upstream of the pre-miR-140 and demonstrate the direct involvement of NFAT3 and SMAD3 on the miR-140 regulatory sequence sites as well as the indirect effect of NFAT5, possibly acting through *WWP2* and *TGF-β*. In turn, the effects of these factors on miR-140 were translated into the cells and impacted a known direct miR-140 target, *IGFBP5*. This study also shows the importance of TGF-β as a factor implicated in the decreased miR-140 expression in human OA chondrocytes, thus contributing to the progression of this disease.

It was first believed that the regulation of intronic miRNAs followed that of their host genes as they are often co-expressed. However, recent reports showed that some intronic miRNAs have their own promoter and that their expression/regulation differs from that of their host gene [26-28]. The differential expression levels of *WWP2* and miR-140 in OA chondrocytes [5,6] led us to believe that this miRNA was controlled by intronic regulatory sequences in addition to the *WWP2* promoter. Other evidence of differential regulation was shown in zebrafish [29] in which miR-140 and *WWP2* were suggested to play distinct roles in cartilage development, as the separate knockdown of *WWP2* and miR-140 caused different effects.

Differential regulation between a miRNA and its host gene may not be a rare event as we have also noted that miR-151 expression, like that of miR-140, is decreased in OA independently of its host gene. However, unlike miR-140, miR-151 has not been identified in miRNA profiling of OA cartilage [30,31], but has been associated with carcinomas [32]. Furthermore, a search through the literature has not revealed any family relationship between the two miRNAs; an *in silico* analysis of the 2 kb sequence located upstream of the mature miR-151 did not reveal any NFAT3 or SMAD3 consensus binding sites, as was the case with miR-140. Although the expression levels of both miR-140 and miR-151 are decreased in OA, their regulation is likely the result of different factors. Thus, the role of miR-151 in OA, direct or indirect, is yet to be determined.

We have identified rsmiR-140 as a regulatory sequence for miR-140 expression independently of its host gene. This sequence is located in a region different from the regulatory sequence identified by Yang *et al*. [33] and the two sequences are likely controlled by different factors. This is in agreement with a search through the miRStart database [34], which revealed two potential transcription start sites (TSS) within the 50,000 bp upstream region of the miR-140 precursor. The first is at position 8,070 bp upstream of the precursor and falls within intron 10 of the *WWP2* gene, upstream of the ATG start of the *WWP2-C* variant as hypothesized by Soond *et al*. [23] and described by Yang *et al*. [33]. It is possible that this TSS is used to initiate *WWP2-C* transcription as miR-140 was reported to be co-expressed with *WWP2-C*[33]. In the Yang *et al*. article [33], however, there are no results showing that the expression of *WWP2-C* is differentially regulated from that of miR-140. The second TSS is located at position 976 bp upstream of the pre-miR-140, thus within the cloned rsmiR-140 sequence, which we have shown to be capable of promoting transcription independently of *WWP2*, unlike the region identified by Yang *et al*. [33].

Another difference from the results of Yang *et al*. [33] is that they found that the C-isoform (variant 2) was abundantly expressed in chondrocytes from mouse limb buds compared to the N- and FL-isoforms which were absent in those cells. This is in contrast to the present findings using human normal and OA chondrocytes in which both the FL and N-isoforms are expressed, but the C-isoform is very weakly or not expressed. The difference between our results and those of Yang *et al*. are not surprising, as the events occurring in mouse limb buds do not necessarily represent what is occurring in human adult articular cartilage.

Mutagenesis has identified functional NFAT3 (GGAAA) and SMAD3 (CAGA) binding sites. Of interest is the opposite direct regulation of miR-140 by NFAT3 (activator) and SMAD3 (inhibitor). Such competition/cooperation between these two factors has previously been reported in different systems, an example being the human c-Myc transcription, activated by NFATs and repressed by TGF-β [35].

This study also identifies NFAT5 as an indirect regulator of miR-140 expression. The differential activity of NFAT5 and NFAT3, although both belong to the same family of transcription factors, is not surprising as they are activated by different factors, have different binding partners and control the transcription of different genes [36-40]. The up-regulation of *WWP2*/miR-140 under hypertonic conditions could occur via the transcription factor Sox9, as *WWP2* and miR-140 were reported to be co-expressed and activated by Sox9 [33] and Sox9 is up-regulated by osmotic stress in human chondrocytes [41]. NFAT5 is also active under isotonic conditions [42]. It was suggested to participate in carcinoma invasion [43] and was found to be a critical regulator of proliferation/survival of synoviocytes in rheumatoid arthritis [44]. Here, we show that, in an isotonic environment, NFAT5 could indirectly down-regulate miR-140 through the stimulation of TGF-β and subsequent activation of SMAD3.

The importance of TGF-β in OA has been recognized and reviewed [45,46] and is reported to have a dualistic role in articular tissue. It protects against cartilage damage by inducing expression of extracellular matrix production, but it also induces osteophyte formation [47,48] and *MMP-13* expression [24,49]. This study reveals another pathway by which TGF-β affects OA chondrocytes: as a down-regulator of miR-140 expression, not only by activating SMAD3 but also by interfering with the translocation of NFAT3. Although the exact mechanism of this interference in chondrocytes remains to be determined, it could occur through a mechanism similar to that described in T-cells in which TGF-β inhibits the phosphorylation of the Tec kinase acting upstream of NFAT activation, thus blocking NFAT translocation into the nuclei [50]. Besides the direct role of TGF-β on miR-140 regulation, it is also known to regulate targets of miR-140, such as *IGFBP-5*[5] and *SMAD3*[51]. Indeed, our group reported that TGF-β increased *IGFBP-5* expression in human OA chondrocytes and Baugé *et al*. [51] found that treating OA chondrocytes for a short period of time up-regulated *SMAD3* expression, but a longer period resulted in a decreased expression. Thus, TGF-β affects many genes and its regulation through miRNAs is part of this complex network.

**Figure 8:**
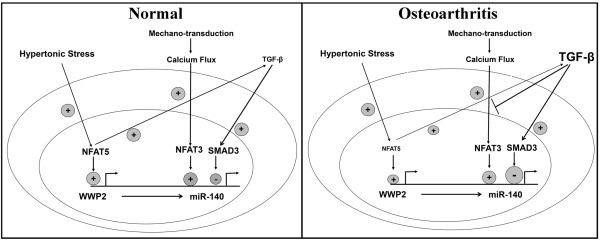
**Hypothesis on the regulation of miR-140 in human normal and osteoarthritic chondrocytes by TGF-β, SMAD3, NFAT3 and NFAT5.** In normal human chondrocytes, mechano-transduction triggers calcium signaling and the subsequent translocation of NFAT3 to the nucleus, where it will up-regulate miR-140. NFAT5, activated under hypertonic stress, up-regulates WWP2 and miR-140 expression. As the levels of TGF-β are low in normal cartilage, the end result will be a positive regulation. In osteoarthritic cartilage, there is a marked increased expression of TGF-β and data showed that the expression levels of NFAT5 are decreased compared to normal human cartilage but those of NFAT3 are stable (data not shown). Thus, TGF-β will increase SMAD3 phosphorylation and directly inhibit miR-140. TGF-β will also indirectly down-regulate miR-140 by interfering with the translocation of NFAT3. Therefore, in osteoarthritic chondrocytes, the NFAT5 contribution will be lower and the TGF-β/SMAD3 negative regulation of miR-140 levels would prevail over the NFAT3 and NFAT5 positive regulation and account for the decrease in this miRNA in these cells. miRNA, microRNA; TGF-β, transforming growth factor β.

Data from this study support the hypothesis that miR-140 expression could be regulated at different levels under normal and OA conditions (Figure 8). Although the thrust of this study was to look at the regulation of miR-140 during a pathological condition, that is, OA, it would also be of interest to evaluate the effects of factors on normal chondrocytes. However, being beyond the scope of the present study, this topic should be explored in another work. Nonetheless, a hypothesis could be as follows. In normal cells, mechano-transduction triggers calcium signaling [52,53] and translocation of NFAT3 to the nucleus, where it will up-regulate miR-140. NFAT5, activated under hypertonic stress, up-regulates *WWP2* and miR-140 expression. As the levels of TGF-β are low in normal cartilage, the end result will be a positive regulation. In OA chondrocytes, the increased expression of TGF-β would activate SMAD3 phosphorylation, thus directly inhibiting miR-140 as well as indirectly down-regulating miR-140 by interfering with the translocation of NFAT3. Therefore, in OA chondrocytes, as the *NFAT5* expression is decreased compared to that in normal chondrocytes (data not shown), the NFAT5 contribution in OA will be lower. However, as TGF-β is increased in OA but the expression of *NFAT3* is similar in normal and OA (data not shown), the negative regulation of miR-140 levels by TGF-β/SMAD3 would prevail over the positive regulation by NFAT3 and would account for the decreased miRNA in these cells.

## Conclusions

In summary, this study is the first to show the important direct roles of NFAT3 and SMAD3 and the indirect role of NFAT5 in miR-140 expression. Moreover, we highlight a new role for TGF-β in OA chondrocytes as a down-regulator of miR-140 expression, resulting in increased expression of miR-140 target genes, thus contributing to this disease process. These data could open up novel avenues in OA therapeutic strategy.

## Abbreviations

(D)MEM: (Dulbecco’s) modified Eagle’s medium; ACR: American College of Rheumatology; ADAMTS: A disintegrin and metalloproteinase with a thrombospondin type 1 motif; BMP2: Bone morphogenetic protein 2; bp: Base pair; BSA: Bovine serum albumin; ChIP: Chromatin immunoprecipitation; DAPI: 4’,6-diamidino-2-phenylindole, dihydrochloride; FAK: Focal adhesion kinase; FCS: Fetal calf serum; GAPDH: Glyceraldehyde 3-phosphate dehydrogenase; IGFBP5: Insulin-like growth factor-binding protein-5; IgG: Immunoglobulin G; MAZ: Myc-associated zinc; miRNA: microRNA; MMP: Matrix metalloproteinase; NFAT: Nuclear factor of activated T-cells; NF-κB: Nuclear factor kappa B; NMP4: Nuclear matrix transcription factor 4; OA: Osteoarthritis; PBS: Phosphate-buffered saline; PCR: Polymerase chain reaction; PTK2: Protein tyrosine kinase; qPCR: Quantitative PCR; rsmiR-140: miR-140 regulatory sequence; SEM: Standard error of the mean; siRNA: Small interfering RNA; SIS3: Specific inhibitor of SMAD3 phosphorylation; SMAD3: Mothers against decapentaplegic homolog 3; SREBF2: Sterol regulatory element binding factor-2; TGF-β: Transforming growth factor beta; TSS: Transcription start sites; WWP2: WW domain containing E3 ubiquitin protein ligase 2.

## Competing interests

The authors declare that they have no competing interests.

## Authors’ contributions

GT, JPP and JMP designed the study. HF, DH, YZ and MK acquired the data. GT, JPP, MK and JMP analysed and interpreted the data. GT, JPP, HF, DH, YZ, MK and JMP participated in manuscript preparation. GT provided statistical analysis. All the authors read and approved the final manuscript.

## Supplementary Material

Additional file 1: Figure S1 Expression as measured by qPCR of WWP2, miR-140, IGFBP5, and TGF-β in OA control chondrocytes; rsmiR-140 activity in control cells from mutagenesis experiments; basal PCR values of NFAT3 and SMAD3 in OA control chondrocytes in ChIP experiments. Click here for file

Additional file 2: Figure S2 Silencing efficiency of siRNAs in human chondrocytes as determined by qPCR and Western blotting. Click here for file

Additional file 3: Figure S3 Basal and induced expression of the WWP2 variants 1 and 2 in human chondrocytes. Click here for file
