# Supplementary figures and images for: NFAT3 and TGF-β/SMAD3 regulate the expression of miR-140 in osteoarthritis
Source: Arthritis Res Ther. 2013 Nov 21;15(6):R197. doi: 10.1186/ar4387 (PMC3978709; doi:10.1186/ar4387)

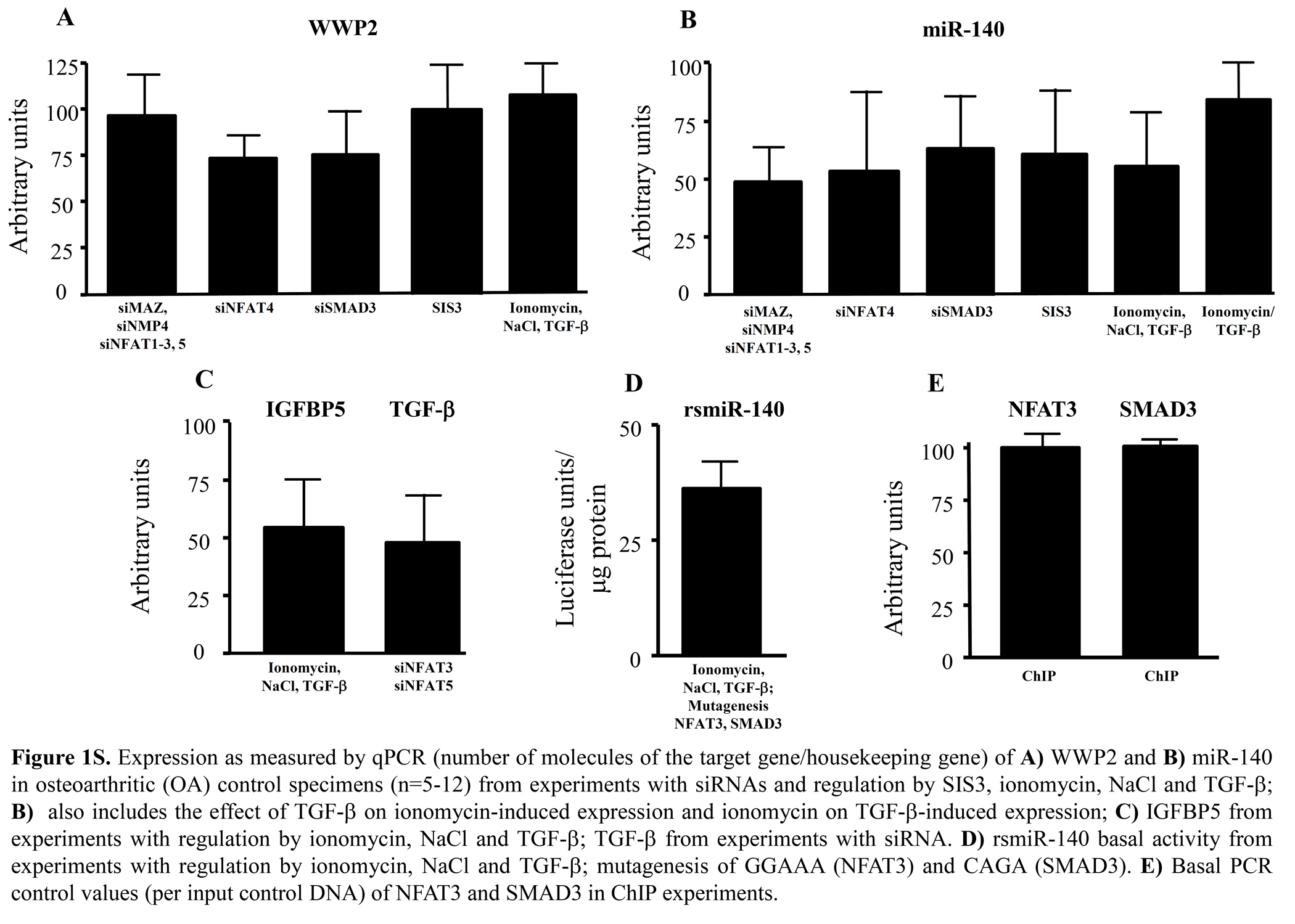

Supplement: Additional file 1: Figure S1 — Expression as measured by qPCR of WWP2, miR-140, IGFBP5, and TGF-β in OA control chondrocytes; rsmiR-140 activity in control cells from mutagenesis experiments; basal PCR values of NFAT3 and SMAD3 in OA control chondrocytes in ChIP experiments. [file ar4387-S1.tiff]

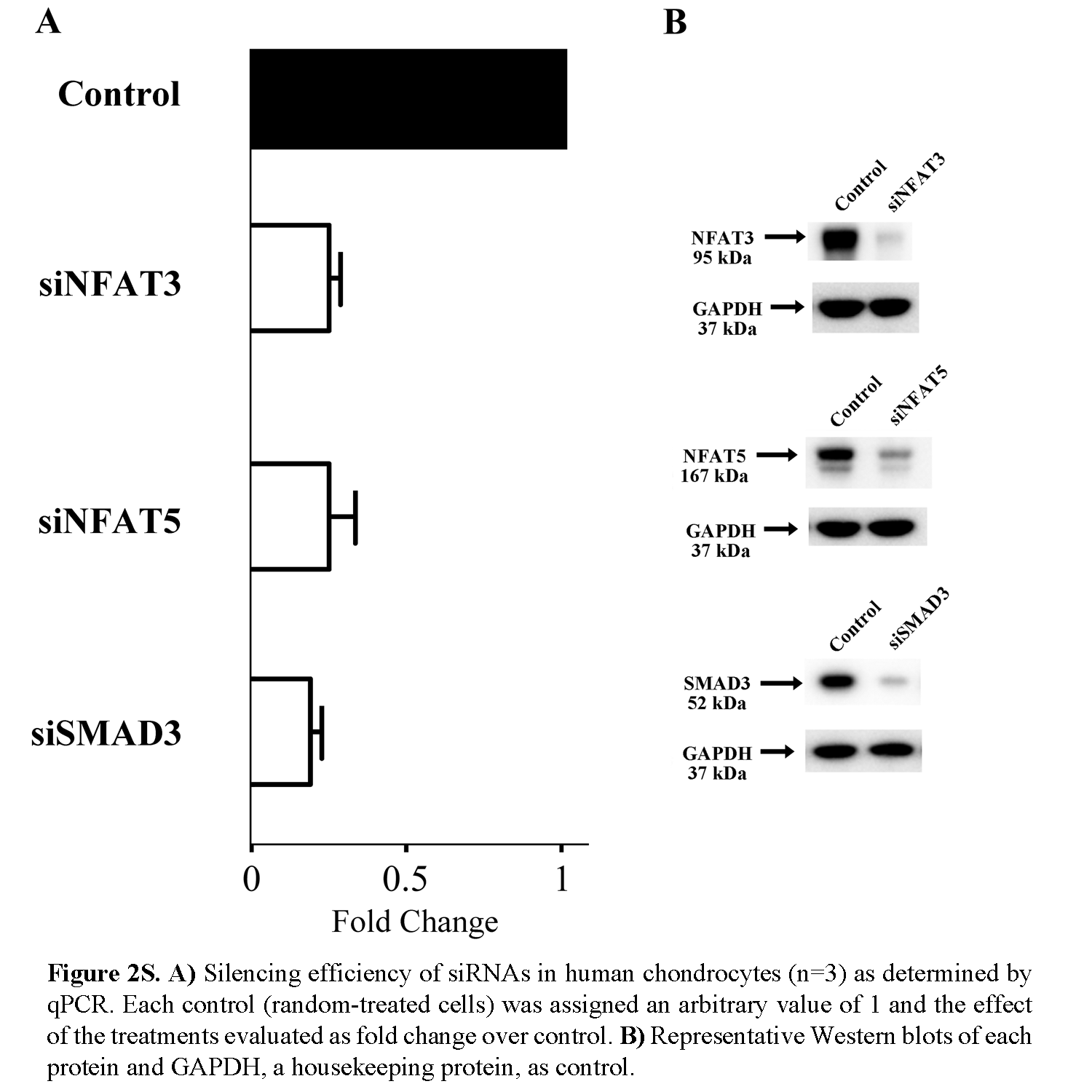

Supplement: Additional file 2: Figure S2 — Silencing efficiency of siRNAs in human chondrocytes as determined by qPCR and Western blotting. [file ar4387-S2.tiff]

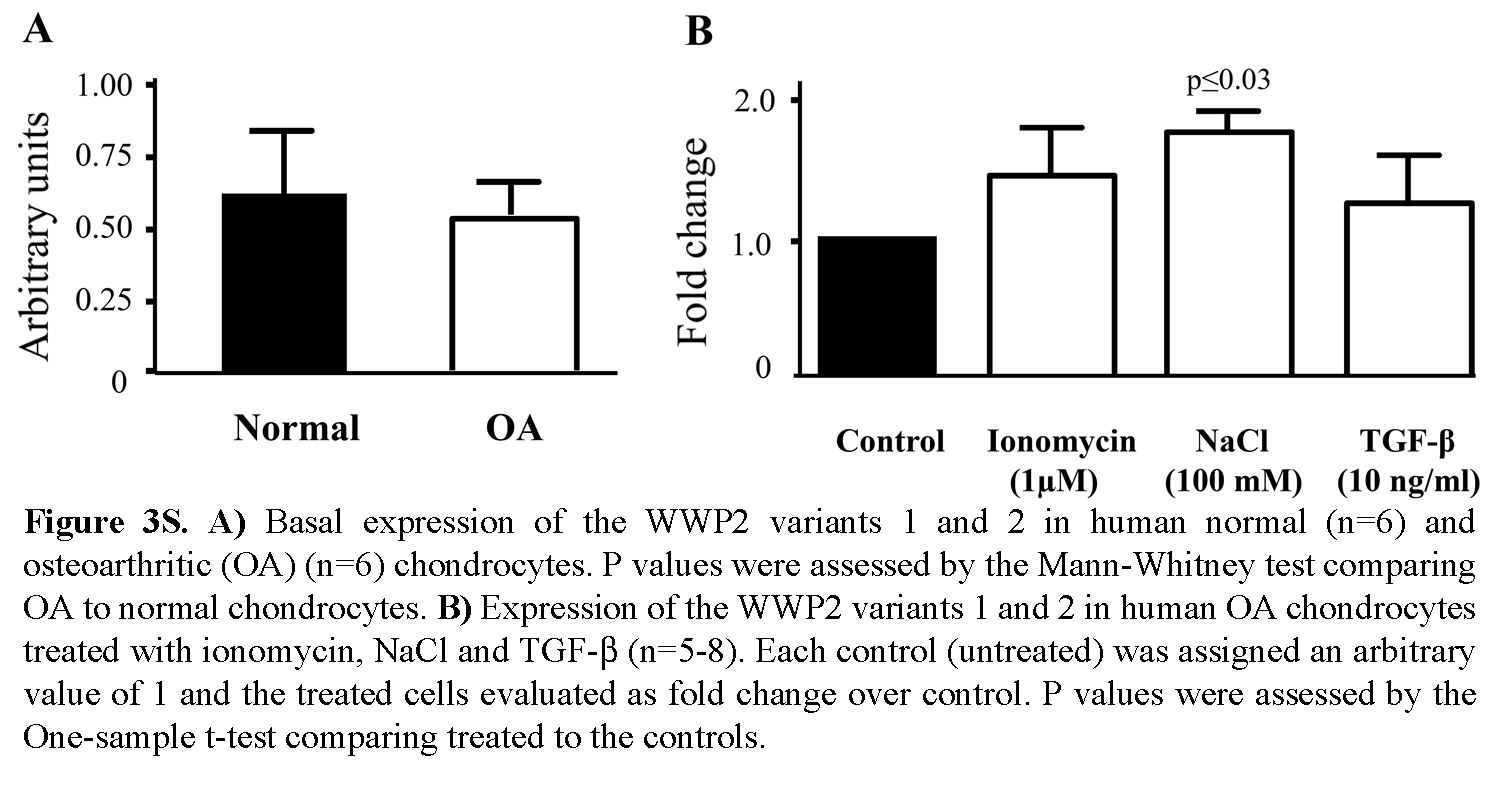

Supplement: Additional file 3: Figure S3 — Basal and induced expression of the WWP2 variants 1 and 2 in human chondrocytes. [file ar4387-S3.tiff]
